# An object localization optimization technique in medical images using plant growth simulation algorithm

**DOI:** 10.1186/s40064-016-3444-2

**Published:** 2016-10-13

**Authors:** Deblina Bhattacharjee, Anand Paul, Jeong Hong Kim, Mucheol Kim

**Affiliations:** 1 Department of Computer Science and Engineering, Kyungpook National University, Daegu, South Korea; 2 The Department of Media Software, Sungkyul University, Anyang, South Korea

**Keywords:** Nature-inspired computing, Plant growth simulation algorithm, Medical image segmentation, Object recognition

## Abstract

The analysis of leukocyte images has drawn interest from fields of both medicine and computer vision for quite some time where different techniques have been applied to automate the process of manual analysis and classification of such images. Manual analysis of blood samples to identify leukocytes is time-consuming and susceptible to error due to the different morphological features of the cells. In this article, the nature-inspired plant growth simulation algorithm has been applied to optimize the image processing technique of object localization of medical images of leukocytes. This paper presents a random bionic algorithm for the automated detection of white blood cells embedded in cluttered smear and stained images of blood samples that uses a fitness function that matches the resemblances of the generated candidate solution to an actual leukocyte. The set of candidate solutions evolves via successive iterations as the proposed algorithm proceeds, guaranteeing their fit with the actual leukocytes outlined in the edge map of the image. The higher precision and sensitivity of the proposed scheme from the existing methods is validated with the experimental results of blood cell images. The proposed method reduces the feasible sets of growth points in each iteration, thereby reducing the required run time of load flow, objective function evaluation, thus reaching the goal state in minimum time and within the desired constraints.

## Background

Various computing techniques inspired from nature has been extensively used in solving problems spanning from optimization, pattern recognition, machine learning, image detection to computer vision. There is hardly any field that is left uninfluenced by the nature-based computing techniques. Image Processing is one such field where recently biomimicry methods are being used invariably. One such application of nature-inspired computation is in the field of medical image processing, especially focused on object localization. In this paper, the problem of object localization in medical images has been solved with the application of the highly efficient plant growth simulation algorithm (PGSA) (Li and Wang 2008) applied to the analysis of white blood cell (WBC) images. WBCs, also known as leukocytes, play a very important role in the diagnosis of a myriad of diseases. In most haematology labs, such cell differential analyses are performed using manual microscopy but this traditional process is not without its limitations. Such analysis require the availability of highly experienced personnel. Due to the substantial possibility of an inter and intra-observer variability in these manual examinations and due to the highly labour intensive routine procedures, new methods for cell analysis are being developed using digital image processing techniques to form better and reliable systems for disease diagnosis. However, high variability of cell shape, edge, localized features, the contrast between cell boundaries and background, cell size and positions have posed as challenges for the efficient object localization during the analysis of such smear images. In this paper, the process of detecting the white blood cells have been undertaken because as per haematology, the WBC tests are tougher and analysing them manually are more difficult as compared to red blood cells. The WBC detection problem has been solved in this article by viewing it as a circle detection problem because WBCs can be approximated as circular in shape. In medical imaging, detecting circular features holds huge significance (Karkavitsas and Rangoussi 2007). As per the existing conventional method of Hough Transform for circle detection in digital images (Muammar and Nixon 1989), an edge detector is used to first find the two necessary and sufficient parameters for circles i.e. the coordinates and the radius of the circle. Upon finding these, the image is averaged over its pixels, followed by filtration for detecting the image peaks. Finally, the image is transformed using a histogram. However, in order to cover all parameters (*x*, *y*, *r*), a lot of memory is required by this method. It also implies a high computational time complexity decreasing its processing speed. Also, the method is not resistant to noise thereby resulting in even lower accuracy (Atherton and Kerbyson 1993). To overcome such a problem, some other approaches based on the Hough transform, for instance the probabilistic Hough transform (Fischler and Bolles 1981; Shaked et al. 1996), the randomized Hough transform (RHT) (Xu et al. 1990), the fuzzy Hough transform, Circular Hough transform with local maximization (Yadav et al. 2014), one-dimensional Hough Transform (Zhou et al. 2014), Hough Transform of curves (Campi et al. 2013) and recently scanline-based hybrid Hough Transform (Seo and Kim 2015) have been proposed with better time complexities but having average memory usage and no noise resistance. In order to overcome the drawbacks of the Hough Transforms, many optimization techniques have been applied to the circle detection problem. These methods have produced much higher accuracy, stability, computational speed and robustness as compared to the discussed Hough Transform as well as other methods like Otsu method based on circular histogram (Wu et al. 2006), WBC identification based on support vector machines (Wang and Chu 2009), and modified transformation methods as proposed in the scientific literatures (Becker et al. 2002; George et al. 2014). These optimization techniques are nature-inspired methods including genetic algorithms (GA) (Ayala-Ramirez et al. 2006), simulated annealing with differential evolution (DE) (Das et al. 2008), harmony search algorithm (Pan et al. 2010, 2011; Cuevas et al. 2012a, b, c), swarm intelligence methods like ant colony optimization based on ant regeneration and recombination to solve the circle detection problem (Chattopadhyay et al. 2008), adaptive bacterial foraging algorithm with adaptive chemotactic step size to facilitate faster convergence (Dasgupta et al. 2008), artificial bee colony algorithm for circle detection (Cuevas et al. 2012a, b, c), clonal selection algorithm for circle detection based on artificial immune system (Isa et al. 2010) and fuzzy cellular neural network (Tong et al. 2005; Wang and Cheng 2007a, b), all of which has been discussed in context of solving the circle detection problem.

Hereunder each optimization method will be discussed one by one and their possible problems that were unaddressed in the cited literature before introducing our proposed scheme. In summary, the genetic algorithm is the most favored computational intelligence model for multi-circle detection so far and has been proven to be more suitable for multi-circle detection problem among other computational intelligence based methods. However, due to the nature of global optimization of genetic algorithm, multi-circle detection requires additional processing. The ideal case would be an algorithm with an inbuilt computational intelligence with a niche adaptability and robustness that only needs to run once like regular deterministic approaches. In Das et al. (2008), simulated annealing and differential evolution has been combined to perform circle detection. Although the method here is robust to noise, it fails to detect circle locations with considerable precision, under both clear and noisy conditions seen from their result samples. In ant colony algorithm (Chattopadhyay et al. 2008), the final circle detection criterion is to threshold the deviation error derived from the detected radius which is the distance between corresponding edge pixels and circle center. This method is essentially a closed loop tracking method, and its performance is questionable when circular shape edges are not enclosed (Chattopadhyay et al. 2008). In Cuevas et al. (2012a, b, c), where the artificial bee colony algorithm has been used, the potential problem is that a lot of memory space would be used if the iteration is set to a large number, but it saves rerun computations. For the fuzzy cellular neural network (Wang and Cheng 2007a, b), the basic limitation is that it takes single inputs where only one WBC is analysed. Moreover, with an exponential increase in the number of iterations the detected circle gets distorted covering the surrounding area, thereby giving more false positives and losing out on the true positives. Also for the adaptive bacterial foraging algorithm in Dasgupta et al. (2008), the method is not inherently capable of detecting multiple circles. In the clonal selection algorithm for circle detection (Isa et al. 2010), both the antigens and antibodies are designed as 10-by-10 images and representations are a binary string which makes it not very practical to process normal resolution images.

In this paper, the detection of WBC has been done by the PGSA. The PGSA is a stochastic evolutionary computation technique based on the natural growth process of a plant towards the global optimal solution—sunlight. Based on plant phototropism, the PGSA regards the feasible region of Integer programming as plant growth environment and evaluates the probability on different growth points according to the changes in the object function. It then grows towards the global optimal solution—light source. The plant grows a trunk from its roots; some branches will grow from the nodes on the branches. This repeats until a plant is formed. The plant branches out through a number of iterations (which can be considered as generations) towards the globally optimal solution, thereby forming an optimal configuration structure that can help it to absorb maximum sunlight for photosynthesis. In the literature (Wang and Cheng 2007a, b), PGSA is compared with other optimization algorithms where the results have shown that the optimal network given by PGSA is the best option as compared to the existing optimization techniques namely genetic algorithms, particle swarm optimization, gradient descent and Tabu search, with a higher rate of accuracy and faster global optimization. As per the analyses, PGSA has the following advantages: (1) the objective function and constraint satisfaction are dealt separately, (2) it does not require any predefined error coefficient, rates of cross-over and mutation therefore resulting in stable solutions, (3) it has a search mechanism with ideal direction and randomness balancing property which is determined by the plant growth hormone (morphactin) concentration and thus finds the global optimal solution quickly. In literatures Luo and Yu (2008), Xu et al. (2012), Lu and Yu (2013), Kumar and Thanushkodi (2013), certain improvements have been carried out on PGSA by studying growth characteristics of plants. The algorithm uses the variable growth rate of the plant vertex to reduce the search time and uses the vertical growth characteristics of the early growth to reduce search space; hence, it is possible to obtain a more optimal solution in less time. Thus, PGSA gives minimum loss while showing greater convergence stability.

The PGSA based circle detector uses three edge points on the image which are randomly selected that represent candidate circles in the edge map of the blood sample image. First, a validation is done to check if these candidates are really present in the image edge map which is generated in the pre-processing stage that will be discussed later in the paper. This is done by calculating the fitness value of such candidates. The better a candidate circle approximates the actual edge circle, the better will be the fitness function value. Hence, the edge map should be accurate and precise enough. Further, the segmentation of the image, which will be mentioned in the pre-processing stage later in the paper, plays an important role to accurately measure the similarity of a candidate circle with an actual WBC. Guided by the values of the new objective function, the set of encoded candidate circles are evolved using the PGSA algorithm so that they can fit into the actual WBC on the image. The approach generates a subpixel detector which can effectively identify leukocytes in real images. PGSA is relatively new, having been introduced in the year 2005 and has never been applied to image processing techniques. This paper aims to apply this highly efficient evolutionary technique towards medical image processing, by proposing a new WBC detector algorithm that efficiently recognizes WBC under different complex conditions while considering the whole process as a circle detection problem.

## Circle detection using PGSA

### Plant growth simulation algorithm (PGSA)

The PGSA is a bionic random algorithm guided by plant phototropism (the ability of a plant to bend towards the light source). The light source is the global optimal solution and the PGSA simulates the mechanism of plant phototropism by assessing the morphactin concentration on the growth points of the plant. This morphactin concentration decides the growth of branches and leaves and is dependent on the intensity of light. PGSA regards the feasible region of Integer programming as plant growth environment and evaluates the probability on different growth points according to the changes in the light intensities taken as the corresponding objective function (Li and Wang 2008; Bhattacharjee and Paul 2016). The algorithm emphasises on a plant system’s method of making decisions which are based on plant’s growth rules and probability models. Biological experiments state the following plant growth laws: First, the node on the plant with a higher morphactin concentration has a greater probability to grow into a branch. Second, the morphactin concentrations on these nodes vary according to the environmental information and the relative positions of these nodes on the plant. If a node has the highest morphactin concentration and hence, if it grows into a branch, the morphactin concentrations of all the remaining plant nodes will be freshly allotted as per the new environment and the just branched node will have a concentration equal to zero.

### Mathematical model for plant growth

According to the probability model of PGSA, *f*(*i*) is the fitness function for the node *i* on the plant to grow in the given environment. The biological laws of plant growth specifies that if the value of *f*(*i*) is smaller, then node *i* has a better environment for growing into a new branch. The mathematical model of PGSA is as follows. Given a root $R_{0}$, a trunk *T* grows from the root. Assuming that there are *n* nodes on the trunk *T* that might provide a more thriving growth environment than the root, i.e. the fitness function $f(R_{Ti}$) < $f(R_{0} )\; \left( {i = 1,2, \ldots ,n} \right),$ the morphactin concentrations $C_{Ti}$ of these nodes are shown in Eq. (1).

1 $$C_{Ti} = \frac{{f\left( {R_{0} } \right) - f\left( {R_{Ti} } \right)}}{{\mathop \sum \nolimits_{i = 1}^{n} \left( {f\left( {R_{0} } \right) - f\left( {R_{Ti} } \right)} \right)}}\quad \left( {{\text{i}} = 1,2, \ldots ,{\text{n}}} \right)$$

**Fig. 1:**
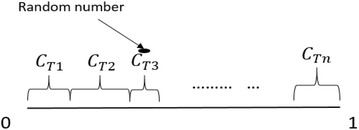
Morphactin concentration space

The above equation shows that the morphactin concentration of a node in a plant is dependent on the growth environment of all the nodes on the plant. A change in the concentration of one node, therefore effects the rest. These concentrations can be imagined as a state space of the interval [0,1] because the $\sum {C_{Ti} }$ = 1. The state space can be shown as in Fig. 1.

Now, from the state space of concentrations a random number is obtained which returns a random concentration $C_{TM}$. The corresponding preferential node $R_{TM}$ takes the priority in the next iteration to branch out. However, the node $R_{TM}$ will grow into a branch only if the random number *β* satisfies the following two Eqs. (2) and (3).

2 $$0 \, \le \beta \le \mathop \sum \limits_{i = 1}^{M} C_{Ti} \quad \left( {M = 1} \right)$$

3 $$\mathop \sum \limits_{i = 1}^{M - 1} C_{Ti} < \beta \le \mathop \sum \limits_{i = 1}^{M} C_{Ti} \quad \left( {M = 2,3, \ldots ,n} \right)$$

After the branch sprouts out, the morphactin concentration of the current node, $R_{TM} ,$ is set to zero and the morphactin concentration of the remaining nodes is reallocated as follows:

4 $$C_{Ti} = \frac{{f\left( {R_{0} } \right) - f\left( {R_{Ti} } \right)}}{{\mathop \sum \nolimits_{i = 1,i \ne M}^{n} \left( {f\left( {R_{0} } \right) - f\left( {R_{Ti} } \right)} \right) + \mathop \sum \nolimits_{j = 1}^{p} \left( {f\left( {R_{0} } \right) - f\left( {R_{bj} } \right)} \right)}}$$

5 $$C_{Ti} = \frac{{f\left( {R_{0} } \right) - f\left( {R_{bj} } \right)}}{{\mathop \sum \nolimits_{i = 1,i \ne M}^{n} \left( {f\left( {R_{0} } \right) - f\left( {R_{Ti} } \right)} \right) + \mathop \sum \nolimits_{j = 1}^{p} \left( {f\left( {R_{0} } \right) - f\left( {R_{bj} } \right)} \right)}}$$

After the reallocation of the concentrations to all the nodes on the plant except $R_{TM}$, the state space of concentrations is again formed with the same interval [0, 1]. Assuming, the newly grown branch *b* has *p* nodes, such that $f(R_{bi}$) < $f\left( {R_{0} } \right) \;\left( {i = 1,2, \ldots ,p} \right),$ again a random number *β* is thrown in the state space and a new node branches out in the next iteration. The new state space has greater number of nodes now, i.e. the nodes previously present (*n* nodes) and the nodes on the new branch *b* (*p* nodes). This growth process stops in the bionic world when the plant has reached its maturity and cannot further branch out.

The PGSA has huge potential to be used in optimization problems. Here, the control parameters are the fitness function [*f*(*i*)], the initial solution (root), search domain of candidate solutions (length of the trunk and branches) and candidate solutions (plant nodes). Further, it has a well-balanced exploration to exploitation ratio (Crepinsek et al. 2013). This method keeps exploring the entire search space with random node selection in the search interval [0, 1]. Although, candidate solutions (nodes) grow in each iteration the search space still remains in the interval [0, 1]. After the exploration, upon the selection of the best candidate solution (preferential node), the morphactin concentrations are reassigned by a neighbourhood like search that assesses the nodes in the vicinity of the just grown branch. The morphactin is not only assigned to the nodes on the new branch by exploitation but also to the previous nodes on the trunk by exploration in a given iteration. This is mainly because the objective function (growth environment) is dependent on the concentration of all the nodes on the plant. Thus PGSA has a well-balanced exploration to exploitation ratio which is necessary for any search optimization algorithm.

**Fig. 2:**
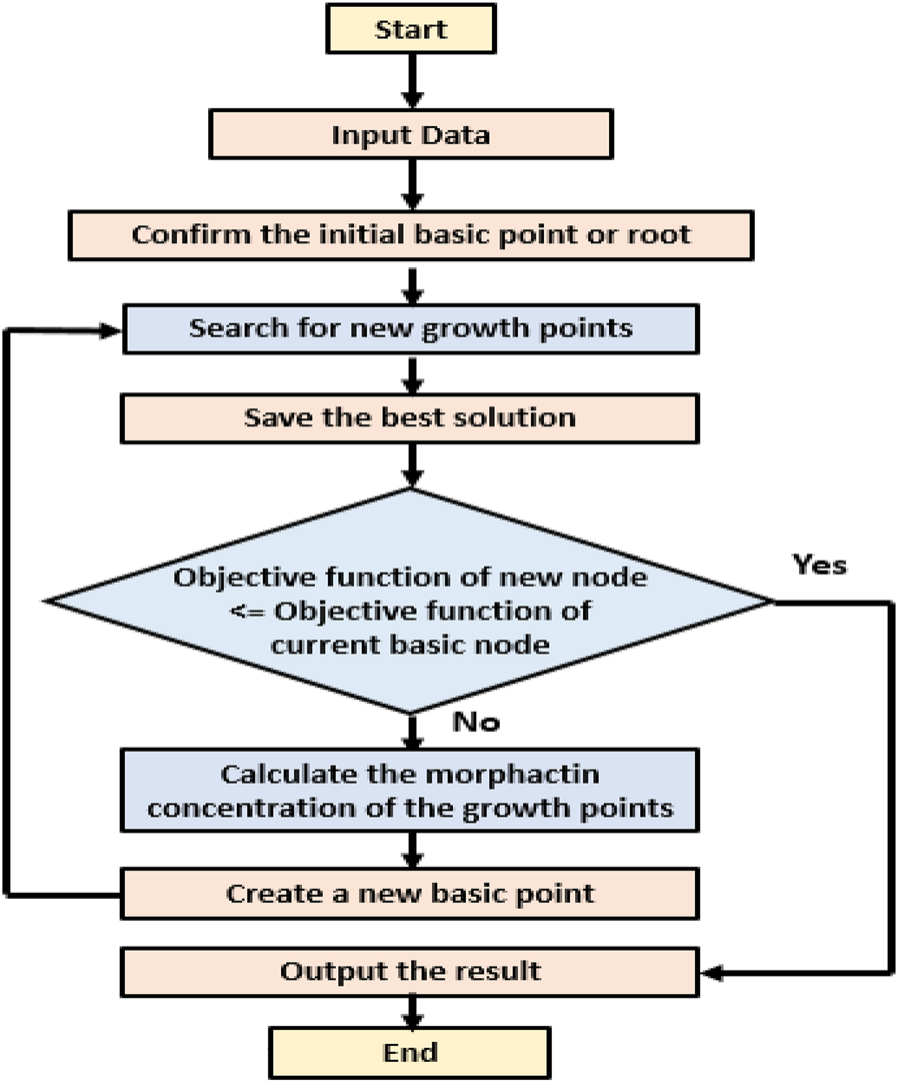
Flowchart of the plant growth simulation algorithm

There have been many algorithms, both traditional and evolutionary, that have been used for circle detection. As has been stated in the background study, all these methods had some disadvantages. PGSA, being inspired from plant phototropism has many advantages that can be used to solve the circle detection problem. The steps of the algorithm has been outlined in Fig. 2.

**Fig. 3:**
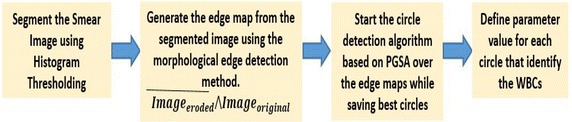
Overview of the process for WBC detection from smear images

**Fig. 4:**
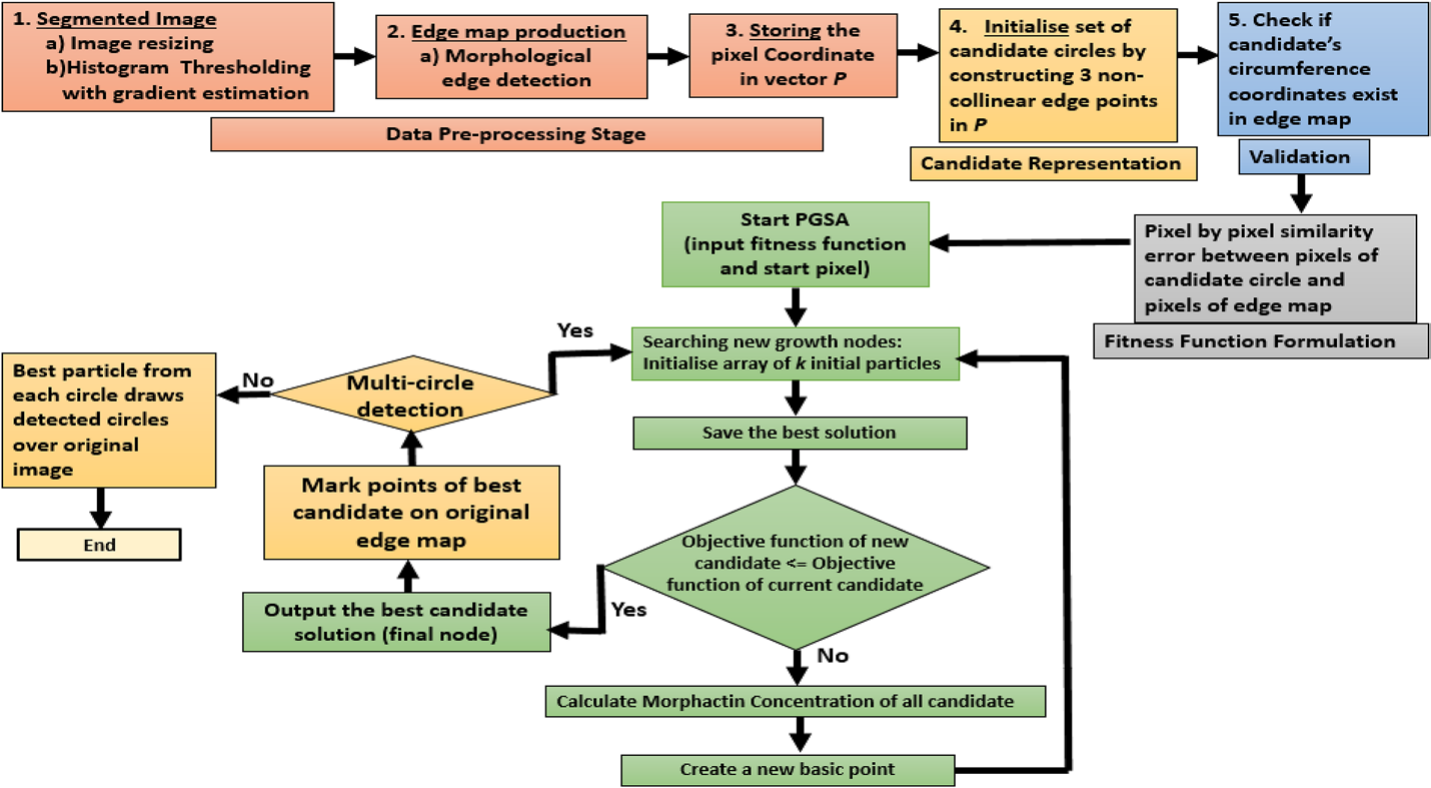
Detailed process for WBC detection from smear images

Our approach is to use the above efficient naturally occurring technique to solve the leukocyte detection problem the overview of which is presented hereunder (Figs. 3, 4).

#### Data pre-processing

To employ the proposed scheme with respect to leukocyte detection, the smear images are pre-processed to obtain two new images. (1) The segmented image and (2) The edge pixel map of the segmented image. For the segmentation pre-processing part, the WBCs are isolated from other structures including red blood cells and the background pixels. Information of colour, brightness, and gradients are used with a corresponding threshold to generate classes to classify each pixel. A histogram thresholding has been incorporated to segment the WBCs.

Now that the segmentation is done, the corresponding edge map is produced. The edge map maintains the total object structure while being just a simple representation of the original image. There many different methods to detect the edges, but for our work the morphological edge detection procedure has been used (Fu and Han 2012; Chandrasiri and Samarasinghe 2014) where erosion followed by inversion of the original image is carried out to ultimately compare it pixel-by-pixel with the original image. This results in the detection of pixels which are present in both the images. This gives the calculated edge map.

Thereafter, the ($x_{i} , y_{i}$) coordinates for every pixel $p_{i}$ defining the image edge is stored in the image edge pixel vector P = $\{ p_{1, } p_{2} , \ldots ,p_{Np} \}$, with $N_{p}$ being the total number of pixels defining the edge of the analysed image.

#### Particle representation for candidate solutions

For the implementation of the algorithm the set of candidate solutions needs to be initialized. For this purpose, the circle candidates need to be constructed using the three non-collinear points on the edge of the circle, previously stored in the vector P. The indices which represent three edge pixel points are grouped assuming that the circle’s contour map connects them together. Let the indices be $e_{i} , i = \left\{ {1,2,3} \right\}$, then the circle *C* passing through these points can be the potential candidate solution for the circle detection problem. *C* = {$p_{{e_{1} }} , p_{{e_{2} }} , p_{{e_{3} }}$}. The centre and radius of the circle *C* are given by the well-known second degree equation as seen in Eq. (6).

6 $$\left( {x - x_{0} } \right)^{2} + \left( {y - y_{0} } \right)^{2} = r^{2}$$

Here $x_{0}$ and $y_{0}$ are computed using

7 $$x_{0} = \frac{\det \left( A \right)}{{4\left( {\left( {x_{j - } x_{i} } \right)\left( {y_{k} - y_{i} } \right) - \left( {x_{k - } x_{i} } \right)\left( {y_{j} - y_{i} } \right)} \right)}},\quad y_{0} = \frac{\det \left( B \right)}{{4\left( {\left( {x_{j - } x_{i} } \right)\left( {y_{k} - y_{i} } \right) - \left( {x_{k - } x_{i} } \right)\left( {y_{j} - y_{i} } \right)} \right)}}$$

where,

$$A = \left[ {\begin{array}{*{20}l} {x_{j}^{2} + y_{j}^{2} - \left( {x_{j}^{2} + y_{j}^{2} } \right)} \hfill & {\quad 2\left( {y_{j} - y_{i} } \right)} \hfill \\ {x_{k}^{2} + y_{k}^{2} - \left( {x_{i}^{2} + y_{i}^{2} } \right)} \hfill & {\quad 2\left( {y_{k} - y_{i} } \right)} \hfill \\ \end{array} } \right]$$

and

$$B = \left[ {\begin{array}{*{20}l} {2\left( {x_{j} - x_{i} } \right)} \hfill & {\quad x_{j}^{2} + y_{j}^{2} - \left( {x_{i}^{2} + y_{i}^{2} } \right)} \hfill \\ {2\left( {x_{k} - x_{i} } \right)} \hfill & {\quad x_{k}^{2} + y_{k}^{2} - \left( {x_{i}^{2} + y_{i}^{2} } \right)} \hfill \\ \end{array} } \right]$$

and

$$r = \sqrt {\left( {x - x_{b} } \right)^{2} + \left( {y - y_{b} } \right)^{2} }$$

Here det(.) stands for the determinant and $b \in \{ e_{1} ,e_{2} ,e_{3} \}$. Therefore, a set of parameters is represented for each circle $[x_{0} ,y_{0} ,r]$ as a transformation *T* for all edge vector indices $e_{1} ,e_{2} ,e_{3}$ which yields:

8 $$[x_{0} ,y_{0} ,r] = T\left( {e_{1} ,e_{2} ,e_{3} } \right)$$

By considering each index as a particle in the search space, the continuous search space is explored by using PGSA for a lookup of circle parameters [$x_{0} ,y_{0} ,r].$

#### Fitness function for the circle detection problem

Before defining the fitness function of the proposed scheme in context of the problem at hand, it is necessary to validate whether the circumference coordinates of the candidate circle *C* exists in the edge image. Upon this validation, the fitness function can be calculated. The coordinates spanning the circle circumference are $J = \{ j_{1} ,j_{2} , \ldots ,j_{N} \}$, where $N$ is the total number of pixel points used for validating the circle coordinates i.e. whether the candidate circle is in the edge map of the image. Thereafter, the fitness function *f*(*C*) is defined as the pixel by pixel similarity error between the above set of pixels J of the circle candidate *C* (particle) and the pixels of the edge map, giving a greater fitness value for a higher resemblance. This function is given as follows,

9 $$f(\varvec{C}) = 1 - \frac{{\mathop \sum \nolimits_{i = 1}^{N} E(j_{i} )}}{N} - \frac{{W_{p} }}{{B_{p} }}$$

where, $E(j_{i} )$ is the expectation function of the presence of the candidate circle pixel at $j_{i}$. Thus, $E(j_{i} )$ has maximum value of 1 if the pixel $j_{i}$ is an edge pixel point. For all other pixel points, the expectation function has a zero value. Also, $W_{p}$ is the amount of white pixel falling inside the candidate circle represented by *C* and $B_{p}$ is the amount of black pixels falling inside *C* (Cuevas et al. 2012a, b, c). These two parameters have been taken into the calculation of the objective function as analysis of smear images cannot be done by directly applying the PGSA algorithm to such images. Smear images present different imaging conditions and staining intensities, which result in noisy edge maps. Thus, in order to use PGSA based circle detector within the context of WBC detection, the fitness function requires these parameters.

#### PGSA implementation

The PGSA has the following steps.

*Step 1* The Canny filter finds and stores the edges in the vector *P* as discussed in the pre-processing step, where *P* contains the set of all edge pixels of the image. The iteration index is set to 1.

*Step 2* *k* initial particles are generated $(C_{a,iteration = 1} ,a \in [1.{\text{k}}])$ in the plant growth environment state space.

*Step 3* The fitness function $f\left( {C_{a,iteration = i} } \right)$ is evaluated to find the best candidate solution like $B_{M2}$ as mentioned in the PGSA discussion in the previous section. This best candidate solution is named as $C^{best} \leftarrow \arg \hbox{min} \{ f(C_{a,iteration = i} )\} .$

*Step 4* As per the PGSA discussed in the previous section, the constraint satisfaction at each of these particles (nodes) is checked, that is their morphactin concentration is calculated as per the Eq. (1). The particle with the higher morphactin concentration has a higher probability to branch out or move to the next iteration as an evolved candidate solution.

*Step 5* The new branch position, which is the new particle’s position is stored and the morphactin concentrations of all the particles are calculated again according Eq. (4) and Eq. (5) except for $C^{best}$ as it has already produced a branch i.e. it is the best solution and hence is the current local optimum solution.

*Step 6* For every new particle, a maximum number of *q* particles are generated as per *j* = 1, 2,…,*q* discussed previously and based on the newly calculated morphactin concentration in *Step 5*, the new best candidate is found on the current generated branch in the previous step. This accounts for a neighbourhood like search for optimal candidate solutions. This process of generating new candidate solutions continues till a better minimized objective function is achieved and stops till there is no improvement in the fitness value of the generated candidate solutions.

*Step 7* The set of all nodes that have branched out are the possible candidate solution with the final node $C^{best}$ being the global best solution and others the local optimal solutions.

*Step 8* From the original edge map, the algorithm marks the points corresponding to $C^{best}$. In case of multi-circle detection it jumps to *Step 2*.

*Step 9* Finally, the best particle $C_{{N_{c} }}^{best}$ from each circle is used to draw (over the original image) the detected circles, where $N_{c}$ is the number of circles detected.

## Experimental results

**Fig. 5:**
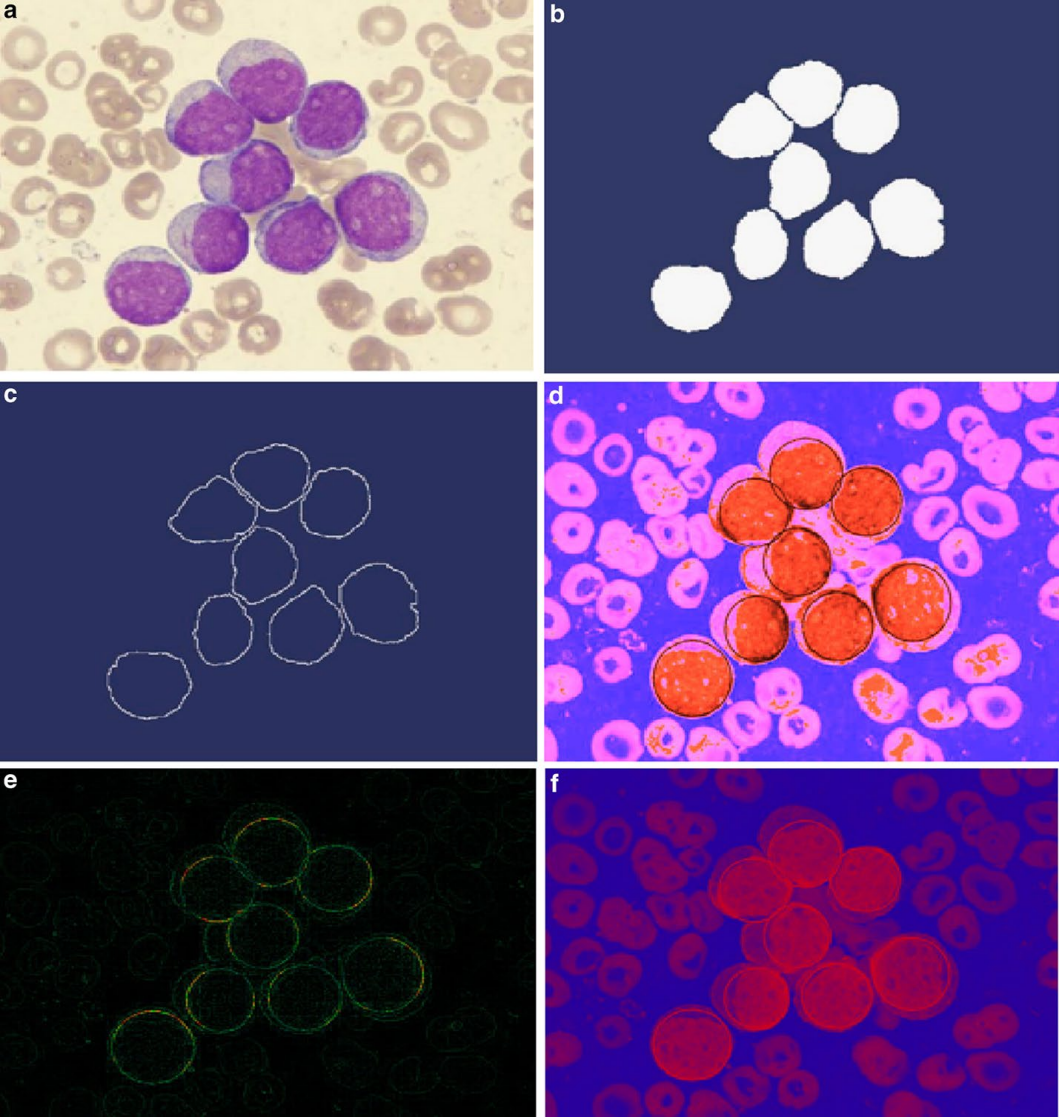
Resultant images of the first test on the application of the WBC detector. **a** Original image, **b** image segmented by histogram transformation, **c** image edge map, **d** heat map of the processed image, **e** the heat map of the detected circles on the leukocytes, and **f** the final result with the detected leukocyte solved in context of the circle detection problem

In order to validate the proposed scheme, experimental tests were carried out and thereafter the algorithm performance was evaluated. The proposed method for the detection of WBC was tested over 80 microscopic images of blood smear with a resolution of 360 × 363 pixels. The precision and sensitivity of the algorithm has been tested under such challenging conditions. The images that were used for the experiments had several deformities, occlusions and overlaps with other images that pose a significant challenge for the circle detection. Figure 5a shows an input blood smear image sample tested with the proposed method for circle detection. Figure 5b shows the segmented WBCs obtained by histogram transforming. Figure 5c is the edge map of the segmented image and Fig. 5d is the heat map of the processed image followed by the heat map of the detected leukocytes as seen in Fig. 5e and finally Fig. 5f gives the detected WBCs.

**Fig. 6:**
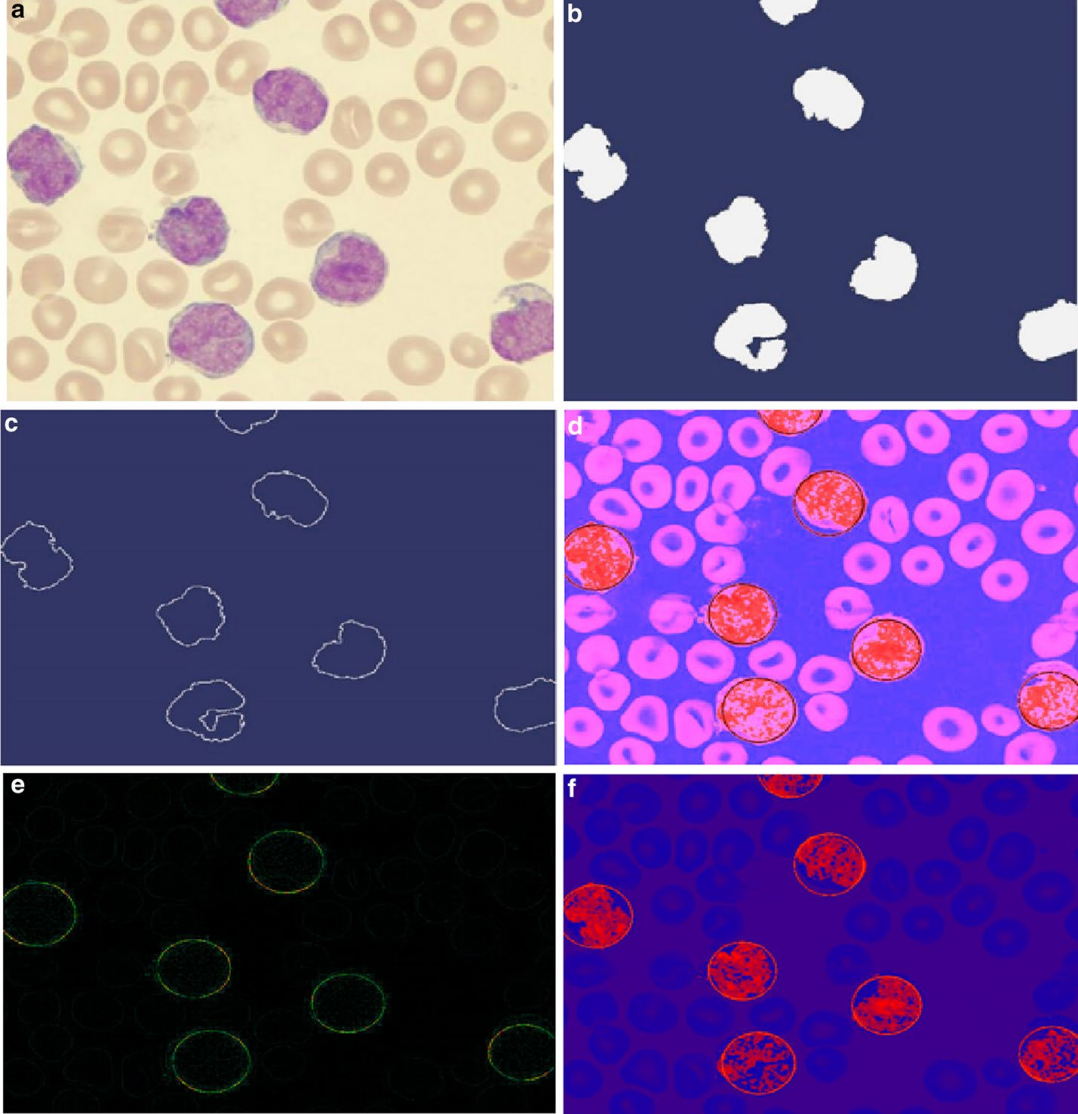
Resultant images of the second test on the application of the WBC detector for a complex and deformed image. **a** Original image, **b** image segmented by histogram transformation, **c** edge map, **d** heat map of the processed image, **e** the heat map of the detected circles on the leukocytes, and **f** the final result with the detected leukocyte solved in context of the circle detection problem

The proposed algorithm is tested on further complicated smear images having highly complex features due to an almost deformed cell. The result is presented in Fig. 6. It represents an example with an image of deformed cells. Clearly, detecting true circles in such complex images becomes tougher. Despite such imperfections, the proposed approach can effectively detect the cells as it is shown in Fig. 6f.

**Fig. 7:**
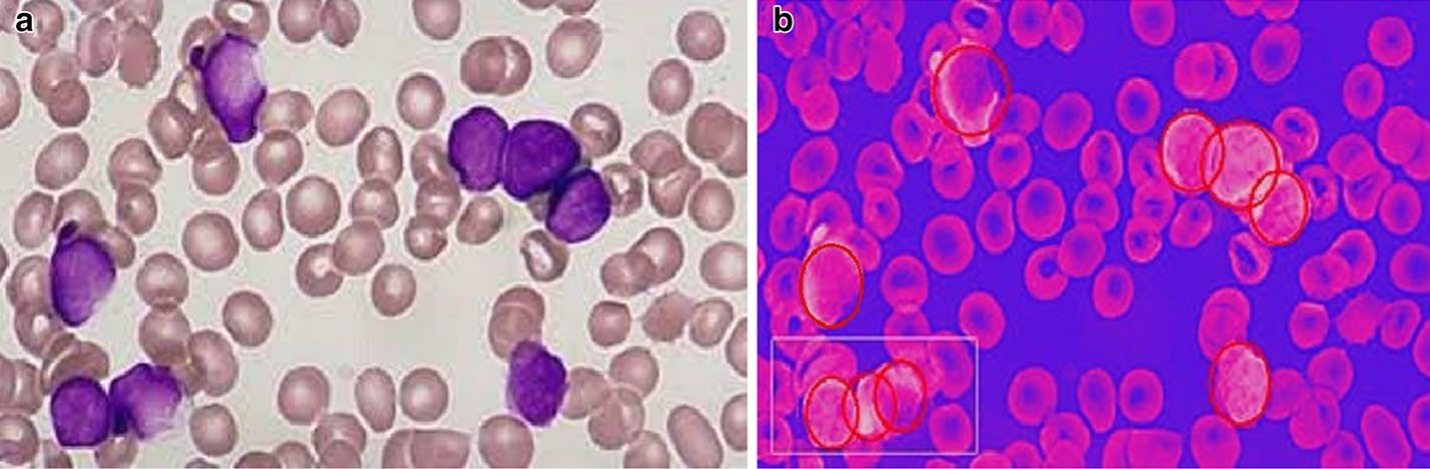
Resultant images of the third test on the application of the WBC detector for a complex image with partially hidden and deformed cells. **a** Original image, **b** result image

Further, the proposed scheme was tested for detecting partial or hidden leukocytes in blood smear images. Detecting partial and hidden leukocytes are utmost challenging for any detection method and thus, experiments were carried out to check if such leukocytes can be correctly detected by the proposed scheme. The result is shown in Fig. 7. Although, the problem is quite complex in nature, PGSA is seen to be quite successful in detecting such leukocytes. However, the leukocytes that were detected were partially still visible with 68 % hidden surface and not for completely hidden leukocytes. Such detections will be part of the future scope of this paper.

Thus, the proposed method can successfully detect damaged, complex and partially hidden leukocytes correctly.

The dataset of smear-blood test images for evaluating the proposed scheme is downloaded from the website Cellavision.com. The dataset includes 80 images from the Cellavision public dataset which were in JPG format of size 360 × 363 pixels, with a resolution of 10 pixels per 1 μm. These images were medically graded and had 463 white blood cells (256 bright leukocytes and 207 dark leukocytes as per the blood smear conditions), all detected by a haematologist—a human medical expert. These numbers were taken as graded standards for all experimentations. For testing the proposed scheme over these images, the true positive rate (known as the number of correctly detected leukocytes over the number of leukocytes detected by the expert) and the false positive rate (known as the number of non-leukocytes that have been wrongly identified as leukocytes over the number of leukocytes which have been actually detected by the medical expert) have been evaluated. The results of the experiments show that the proposed method, achieves 98.28 % leukocyte True Positive Rate with 1.72 % False Positive Rate, and is therefore, arguably better than the other existing methods. To establish this statement, the proposed scheme has been further discussed and compared with other existing methods in context of the leukocyte detection problem as a circle detection problem hereunder.

## Discussion

As this circle detection problem can be considered as a binary classification problem, where the detections can be classified as either positive or negative, the classification can be represented as a confusion matrix which has the following four classes. (1) True Positives for circles correctly detected as positives; (2) False Positives are wrongly detected circles as positives; (3) True Negatives are non-circles correctly detected as such; (4) False Negatives are positive circles incorrectly labelled as non-circles (Parikh et al. 2008, Davis and Goadrich 2006). According to the problem of circle detection, the circle can be either correctly detected (true positive), wrongly detected (false positive) or not detected at all (false negative). Thus there is no true negative in a circle detection task. A true negative condition would have arisen if there isn’t a circle in the image and the algorithm also hasn’t detected it. As it is a detection problem, non-detections of non-circles are not taken into consideration. Further, based on the confusion matrix, the parameters of true positive, false positive and false negative have been taken to evaluate the performance indices like true positive rate, false positive rate, false discovery rate and positive predictive value for further analysis of the proposed scheme. However, other performance indices like specificity and negative predictive value have not been used as they require true negatives by definition for the calculations (Parikh et al. 2008). As mentioned above, the dataset of 80 images from Cellavision Reference Library was used to identify both bright and dark leukocytes using our proposed scheme. The total number of detected leukocytes, wrongly detected leukocytes and undetected leukocytes have been shown in the Table 1 below. The detection rate and other performance indices in the confusion matrix have been compared among four algorithms. The images from the Cellavision dataset are processed by hough transform (traditional method), modified genetic algorithm with ant colony optimization (both GA and swarm intelligence method are analysed here), fuzzy cellular neural network (which is a member of neural networks) and the proposed method of PGSA; as discussed previously in the background section. All the four algorithms are applied by maintaining their own settings. Thus the images were processed by making no further adjustments to the existing methods, thereby facilitating the performance comparison of all the methods over different blood smear images with varying background and target object conditions. These resultant images are then checked with medically graded standards by a medical expert.

**Table 1:** Comparative performance of HT, FCCN, GA+ACO and PGSA for leukocyte detection with respect to true positive rate, false positive rate, false discovery rate and positive predictive value

Leukocyte type	Method	Leukocytes detected (true positives)	Missing leukocytes (false negatives)	Missing leukocytes (false negatives)	True positive rate (%)	False positive rate (%)	False discovery rate (%)	Positive predictive value (%)
Bright leukocyte (256)	HT	135	121	67	46.85	30.18	59.90	66.83
	FCCN	206	50	55	78.83	24.77	19.16	78.93
	GA + ACO	217	39	42	83.78	18.92	15.06	83.78
	PGSA	242	14	10	98.01	1.99	5.56	96.03
Dark leukocyte (207)	HT	100	107	54	48.04	26.47	69.48	64.94
	FCCN	168	39	49	81.37	24.02	17.97	77.42
	GA + ACO	183	24	38	88.72	18.63	10.86	82.81
	PGSA	202	5	6	98.55	1.45	2.40	97.12
Overall (463)	HT	235	228	121	47.42	28.40	64.04	66.01
	FCCN	374	89	104	80.05	24.41	18.62	78.24
	GA + ACO	400	63	80	86.15	18.78	13.13	83.33
	PGSA	444	19	16	*98.28*	*1.72*	4.13	96.52

Italic values signifies the % of true and false positive

**Fig. 8:**
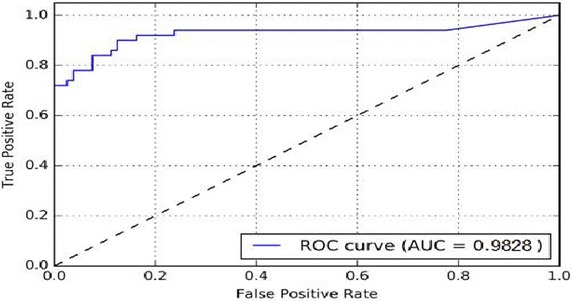
ROC curve of the true positive rate versus the false positive rate in the proposed method

It has been already seen that as far as the detection comparison is concerned the PGSA technique fairs extremely well with a detection rate of 98.28 % and is better when compared to other leukocyte detection algorithms. The detection rate is depicted from the ROC curve of the True Positive Rate or the Detection Rate versus the False Positive Rate as shown in Fig. 8.

**Fig. 9:**
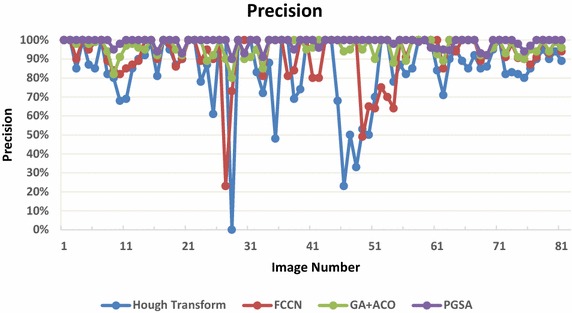
The precision measure for the detection of true positives by all the four algorithms

**Fig. 10:**
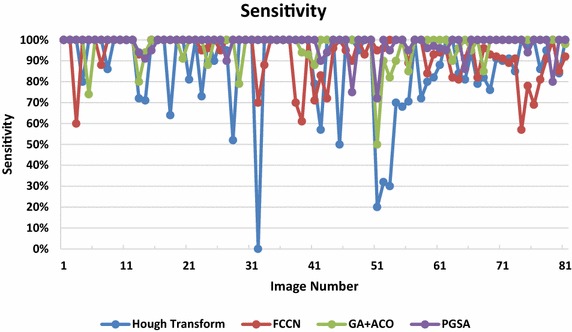
The sensitivity graph for the four algorithms

Thereafter all the results are analysed with the help of statistically calculated precision and sensitivity graphs as shown in Figs. 9 and 10. The algorithm is tested out and evaluated for its efficiency based on two parameters named as precision and sensitivity as defined below.

10 $$Precision = \frac{correctly\;detected\;circles}{correctly\;detected\;circles + wrongly\;detected\;circles}$$

11 $$Sensitivity = \frac{correctly\;detected\;circles}{correctly\;detected\;circles + undetected\;circles}$$

Under ideal conditions, the precision and sensitivity both are 100 %, thus the circle detection technique under such scenario would be able to find all the circles in the image without any false positives or un-detected circles. But under normal conditions precision and sensitivity is seen to decrease because when detecting such circles from the images the circle detection method has to find both the centre of the circle as well as the radius of the circle.

Precision is a measurement of the rate of correct detection over all detected circles. A perfect situation arrives when there is a straight line all along 100 % of y-axis in Fig. 6 for all the images. It is apparent that Hough Transform based method has the worst results, which is not beyond expectation. A completely extreme case is a 0 % precision, implying no detection at all. Regular Hough Transform suffers a number of false detections with many images. The Modified Genetic Algorithm with Ant Colony Optimization and PGSA are seen to achieve correct detection above 90 % with small standard deviation, which are superior to the Fuzzy Cellular Neural Network (FCCN) and evidently the conventional Hough Transform. The possible problem with the FCCN is that it requires a lot of computational time and memory to train its network and the detection is achieved over a large number of generations. Also, as shown in Cuevas et al. (2012a, b, c), when the number of iterations increases, the possibility to cover other structures increases too. Thus, if the image has a complex background like in smear images, the method gets confused because of which finding the correct contour configuration from the gradient magnitude becomes highly difficult.

Sensitivity evaluates the detection rate over all existing circular shapes. On one hand, a low sensitive setting would result in good precision numbers because detected circles must be true and distinctive, while sensitivity rate would be low because non-perfect shapes are missing. On the other hand, high sensitivity means reducing missing incidence but increasing the false detection rate at the same time. The PGSA model initializes nodes on all edge segments, which gives an almost perfect 100 % overall sensitivity, due to every edge segment being checked for constraint satisfaction during each iteration as seen in Fig. 10. Additionally, with the inherent randomness and ideal load balancing capabilities to give optimal configurations, this stochastic method converges very fast as well (Table 2).

**Table 2:** Statistics of sensitivity for the four analysed algorithms

Approach	Maximum	Minimum	Mean	SD
Hough transform	1	0	0.87	0.198
FCCN	1	0.56	0.93	0.104
GA + ACO	1	0.5	0.97	0.075
PGSA	1	0.73	0.98	0.049

**Fig. 11:**
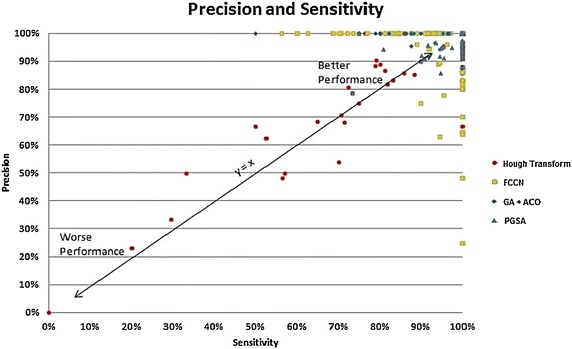
The precision and sensitivity graph of the four algorithms

In Fig. 11, the results of both the precision and sensitivity have been combined at the same time from the previous two figures. Under the ideal situation, the result would be that all the values would have been centred at the top right corner giving 100 % detection rate. In Fig. 11, the line of y = x indicates an equal probability detection rate based on hit and trial where the probability of such detections become 0.5 or 50 %. Thus, after both precision and sensitivity were combined, the data samples were seen to be scattered for the algorithm with the least satisfactory performance while the data points were seen to incline towards the upper right corner or 100 % detection rate with an improved algorithm like FCCN, Genetic Algorithm with Ant Colony Optimization and PGSA, in that order. This shows the advantage of PGSA model over the other methods.

**Fig. 12:**
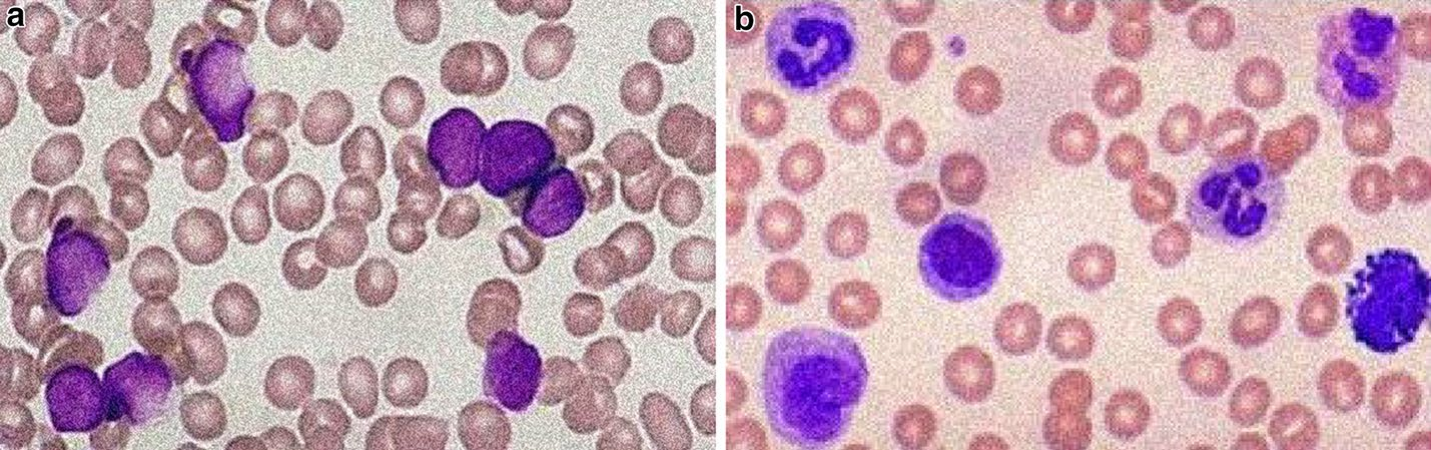
Examples of noisy image contaminated with **a** Gaussian noise at σ = 15 and **b** salt and pepper noise at 15 %, used in the experiment to measure the noise-resistance of the various methods

In addition to the above performance measures, the proposed algorithm was also tested for its resistance towards noise. Most of the blood smear images are noisy because of defects or noise in the system that acquires such images, the haematology equipment or other factors. Thus, the circle detection task also depends on how noise-resistant the applied algorithm is. In order to test this performance characteristic of the proposed algorithm the images from the used dataset were corrupted using the (1) Gaussian noise, (2) Salt and pepper noise, which are the most pedestrian noise found in smear images of blood samples. All the 80 images containing 463 leukocytes (both bright and dark) were corrupted with these two noise types in varying levels and were analysed by applying the four algorithms. The Gaussian noise level was used at σ = 10 and σ = 15 and the salt and pepper noise level was used at 10 and 15 %. Examples of such corrupted images used for the noise-resistance experiment are shown in Fig. 12. The performance of these algorithms are shown in Tables 3 and 4 respectively.

**Table 3:** Comparative performance of leukocyte detection over 80 images contaminated by various levels of gaussian noise

Noise level	Method	Leukocyte detected (true positives)	Missing Leukocyte (false negatives]	Wrongly detected leukocyte (false positives)	True positive rate (%)	False positive rate (%)	False discovery rate (%)	Positive predictive value (%)
Gaussian noise 463 leukocytes σ = 10	HT	206	257	77	40.37	18.07	90.81	72.79
	FCCN	343	120	71	72.53	16.67	28.99	82.85
	GA + ACO	335	128	65	70.66	15.26	32.00	83.75
	PGSA	431	32	21	93.19	4.93	7.08	95.35
Gaussian noise 463 leukocytes σ = 15	HT	177	285	106	33.57	24.88	98.77	62.54
	FCCN	315	148	89	65.96	20.89	36.63	77.97
	GA + ACO	298	165	102	61.97	23.94	41.25	74.50
	PGSA	414	49	32	89.20	7.51	10.99	92.83

**Table 4:** Comparative performance of leukocyte detection over 80 images contaminated by various levels of salt and pepper noise

Noise level	Method	Leukocyte detected (true positives)	Missing leukocyte (false negatives)	Wrongly detected leukocyte (false positives)	True positive rate (%)	False positive rate (%)	False discovery rate (%)	Positive predictive value (%)
Salt and pepper Noise level 10 % 463 leukocytes	HT	182	281	114	34.74	26.76	94.93	61.49
	FCCN	304	159	106	63.38	24.88	38.78	74.15
	GA + ACO	284	179	118	58.68	27.70	44.53	70.65
	PGSA	424	39	30	91.55	7.04	8.59	93.39
Salt and pepper Noise level 15 % 463 leukocytes	HT	135	328	120	23.71	28.17	128.63	52.94
	FCCN	274	189	78	56.34	18.31	53.69	77.84
	GA + ACO	218	245	123	43.19	28.87	71.85	63.93
	PGSA	408	55	35	87.79	8.21	12.42	92.10

Thus, even under noisy conditions the PGSA is the most robust method to detect the leukocytes with the best detection rate, best positive predictive value, least false positive rate and the least false discovery rate.

## Conclusion

In this paper, a new bionic random search algorithm has been proposed that makes use of the objective function’s value as an input to the learning model while simulating a plant’s phototropism for the automatic detection of WBCs that are embedded into complicated, obscure and cluttered smear images by considering the WBC detection problem as a circle detection problem. The PGSA has been applied to solve this circle detection problem which gives the location of the WBCs in the images using three non-collinear edge points on the segmented edge map of the image as candidate circles. The resemblance of the encoded candidate circles to the actual WBC is evaluated by the objective function which uses the edge map and segmentation results for calculating the resemblances. Based on the calculated value of the objective function, the set of encoded candidate circles (branch nodes) are evolved by using the PGSA so that they can fit into the actual blood cells that are contained in the edge map. The experimental results and the performance of the PGSA has been compared with other existing WBC detection algorithms which demonstrate the high performance of the proposed method in terms of detection accuracy, precision, and sensitivity and also under noisy conditions. Although, there has been quite some research done to solve the circle detection problem when processing images, it has not been applied in the context of medical image processing. Moreover, PGSA has never been applied to solve such a problem. This evolutionary algorithm is highly efficient and is new to the field of computing intelligence. Thus, it offers a lot of scope for applications, implementations and further extension of this algorithm.
